# Underwater photogrammetry for close‐range 3D imaging of dry‐sensitive objects: The case study of cephalopod beaks

**DOI:** 10.1002/ece3.7607

**Published:** 2021-05-03

**Authors:** Marjorie Roscian, Anthony Herrel, Raphaël Cornette, Arnaud Delapré, Yves Cherel, Isabelle Rouget

**Affiliations:** ^1^ Centre de Recherche en Paléontologie‐Paris (CR2P) Muséum National d'Histoire Naturelle CNRS Sorbonne Université Paris France; ^2^ Mécanismes Adaptatifs et Evolution (Mecadev) Muséum National d'Histoire Naturelle CNRS Bâtiment d'Anatomie Comparée Paris France; ^3^ Institut de Systématique, Évolution, Biodiversité (ISYEB) Muséum national d'Histoire naturelle CNRS Sorbonne Université EPHE Université des Antilles Paris France; ^4^ Centre d'Etudes Biologiques de Chizé UMR7372 CNRS‐La Rochelle Université Villiers‐en‐Bois France

**Keywords:** 3D models, cephalopod beaks, dry‐sensitive material, micro‐CT scanning, micro‐photogrammetry, underwater photogrammetry

## Abstract

- Technical advances in 3D imaging have contributed to quantifying and understanding biological variability and complexity. However, small, dry‐sensitive objects are not easy to reconstruct using common and easily available techniques such as photogrammetry, surface scanning, or micro‐CT scanning. Here, we use cephalopod beaks as an example as their size, thickness, transparency, and dry‐sensitive nature make them particularly challenging. We developed a new, underwater, photogrammetry protocol in order to add these types of biological structures to the panel of photogrammetric possibilities.
- We used a camera with a macrophotography mode in a waterproof housing fixed in a tank with clear water. The beak was painted and fixed on a colored rotating support. Three angles of view, two acquisitions, and around 300 pictures per specimen were taken in order to reconstruct a full 3D model. These models were compared with others obtained with micro‐CT scanning to verify their accuracy.
- The models can be obtained quickly and cheaply compared with micro‐CT scanning and have sufficient precision for quantitative interspecific morphological analyses. Our work shows that underwater photogrammetry is a fast, noninvasive, efficient, and accurate way to reconstruct 3D models of dry‐sensitive objects while conserving their shape. While the reconstruction of the shape is accurate, some internal parts cannot be reconstructed with photogrammetry as they are not visible. In contrast, these structures are visible using reconstructions based on micro‐CT scanning. The mean difference between both methods is very small (10^−5^ to 10^−4^ mm) and is significantly lower than differences between meshes of different individuals.
- This photogrammetry protocol is portable, easy‐to‐use, fast, and reproducible. Micro‐CT scanning, in contrast, is time‐consuming, expensive, and nonportable. This protocol can be applied to reconstruct the 3D shape of many other dry‐sensitive objects such as shells of shellfish, cartilage, plants, and other chitinous materials.

## Graphical abstract

We propose a new close‐range underwater photogrammetry protocol adapted to small dry‐sensitive objects. We used cephalopod beaks as a case study because they combine several problems for 3D imaging. The comparison between photogrammetry and microtomography shows a high accuracy of the models reconstructed by photogrammetry, allowing various applications for quantitative analysis.

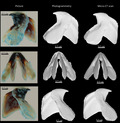

## 1 INTRODUCTION

Quantifying the complexity and the variability of biological objects has been a long‐standing challenge, yet it is critical to understand the evolution of phenotypic diversity (Haleem & Javaid, 2019; Houle et al., 2010; Pears et al., 2012; Sansoni et al., 2009; Vázquez‐Arellano et al., 2016). Technical advances in 3D imaging over the last decades, including 3D microscopy, surface scanning, and X‐ray computed tomography have renewed our visualization and shape modeling capabilities, pushing the boundaries of structure detection and increasing spatial resolution (Carlson et al., 2003; Lidke & Lidke, 2012; Ziegler et al., 2010; Mathys et al., 2019; Pears et al., 2012; Richardson, 2013; Tafforeau et al., 2006; Walter et al., 2010). Numerous 3D imaging techniques are currently available, including photogrammetry, laser scanning, magnetic resonance imaging (MRI), and computed tomography, among others (Pears et al., 2012; Remondino & El‐Hakim, 2006). Consequently, image acquisition methods are now available for most biological objects but they depend on technical constraints inherent to the biological sample under study (composition, texture, size…), resolution requirements, and the time and expense that can be devoted to analyses.

X‐ray computed tomography (here micro‐CT scanning) is nowadays routinely used for the imaging of biological structures as (a) it allows the visualization of internal and external structures of a large variety of objects ranging from soft tissues (with the use of contrast agents; see Gignac & Kley, 2018; Gignac et al., 2016; Metscher, 2009) to fossil specimens, (b) it is largely nondestructive, and (c) it requires little sample preparation. For the imaging of soft tissues that consist largely of water, magnetic resonance imaging (MRI) is an alternative (Digital Fish Library, 2009; Zanette et al., 2013) and is widely used in medicine, physiology, and neurobiology (Rasmussen et al., 2010). However, this method does not allow the accurate imaging of “dry” objects such as bones and it typically has a longer acquisition time and lower spatial resolution than micro‐CT scanning (Ziegler et al., 2010). For external 3D surfaces, surface scanners and photogrammetry have the advantage of being easier to implement than the previous two methods. The resolution can be on the order of a few microns depending on the type of surface scanner and the acquisition time is low, ranging from a few minutes to about 10 min, depending on the desired resolution (Fourie et al., 2011; Skarlatos & Kiparissi, 2012). By using structured light scanners, the color of the object can be preserved, allowing the analysis of patterns as well (skin, pigmentation etc.). This specificity is also provided by photogrammetry, which is the easiest and cheapest method while at the same time accurate and reliable to implement, thus allowing the 3D analysis of a large number of objects. As photogrammetry is based on the identification of points on overlapping pictures of the object and their projection into three dimensions, photogrammetry is well suited for the reconstruction of textured objects but it performs rather poorly with smooth or shiny objects (Guery et al., 2017; Kraus & Waldhäusl, 1997; Luhmann et al., 2020).

The abovementioned techniques are widely used in biological studies under laboratory conditions, or even in the field (e.g., laser scanning and photogrammetry), yet most often concern the imaging of dry structures. For wet specimens or objects sensitive to dehydration, laser scanning cannot be used as the object will deform during scanning due to progressive dehydration over time. Micro‐CT scanning is, however, efficient for imaging specimens in a liquid environment (Broeckhoven & Plessis, 2018). Whereas micro‐CT scanning is time‐consuming and relatively expensive when a large quantity of objects needs to be imaged, underwater photogrammetry is a well‐known alternative method that is cheap, reproducible, and portable. Developed during the sixties, it has been used in several contexts such as the mapping of marine habitats (Abadie et al., 2018), estimating the body mass of marine mammals (Bell et al., 1997; Christiansen et al., 2019), determining the growth rate of biological structures (Drap, Merad, Mahiddine, Seinturier, Gerenton, et al., 2013; Kikuzawa et al., 2018; Lange & Perry, 2020; Olinger et al., 2019), or in marine archaeology (Drap et al., 2006; Drap, Merad, Mahiddine, Seinturier, Peloso, et al., 2013). Underwater photogrammetry faces specific constraints related to the aquatic environment, notably light variation, scattering effects, turbidity (Agrafiotis et al., 2018; Bianco et al., 2013; Ormestad et al., 2013), and refractive effects (Maas, 2015). Over the years, techniques and cameras have improved and even close‐range photogrammetry is now used for the imaging of biological objects in their natural environment (Olinger et al., 2019). Surprisingly, this technique has been only rarely used under controlled laboratory conditions, possibly due to the difficulty in resolving the trade‐off between imaging with enough accuracy while maintaining time and cost low.

Cephalopods beaks combine many of the difficulties associated with the imaging of objects sensitive to drying and 3D modeling. Their shape is complex, the walls of the structure thin, the texture is smooth and often homogeneous, and these objects can show a gradient from transparent (untanned) to dark brown (heavily tanned) (Miserez et al., 2008). They cannot be imaged outside of water as they deform extremely quickly due to drying. Given these difficulties, no 3D models representing these structures exist to date with the exception of the 3D model created by Uyeno and Kier (2005) using serial histological sections. The jaws of cephalopods consist of a complex mixture of chitin, water, and proteins (Miserez et al., 2010; Tan et al., 2015). Due to their composition, cephalopod beaks are among the rare structures durably preserved in stomachs of predators such as whales, predatory fish, and sea birds. They provide species‐specific information allowing their identification (Clarke, 1986) and may provide information on trophic ecology through stable isotope analyses (Cherel, 2020; Cherel & Hobson, 2005). Such access to accurate 3D models would be of interest to a wide range of researchers. As micro‐CT scanning is time‐consuming and expensive, and often fails to capture the extremely thin nontanned parts of the beak in small specimens, underwater photogrammetry appears to be an interesting alternative to create undeformed 3D models of cephalopod beaks. Here, we present a close‐range underwater photogrammetry protocol adapted to dry‐sensitive objects, validated by a quantitative comparison between these photogrammetric models with ones obtained through micro‐CT scanning.

## 2 MATERIAL AND METHODS

### 2.1 Sample

**FIGURE 1:**
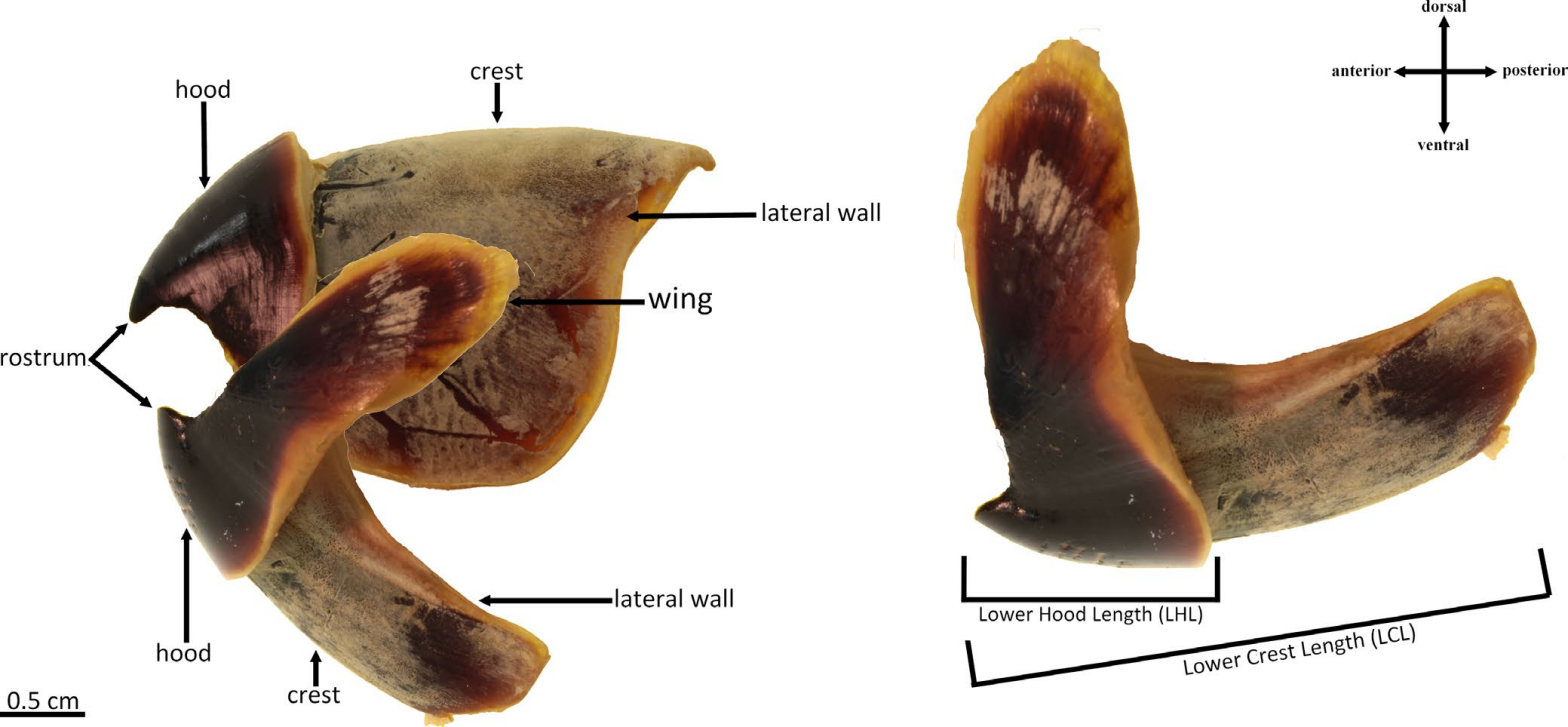
Anatomy of cephalopod beaks, here *Octopus vulgaris* beak (photographed) on the left, and common measurements on the lower beak on the right using the terminology proposed by Clarke (1986)

Cephalopod beaks are composed of two distinct parts: an upper beak and a lower beak. Both are composed of a hood on the anterior part as well as a rostrum and wings (Clarke, 1962). Posteriorly, the beak bears lateral walls and a crest on top (Figure 1). Common measurements used for the identification of the lower beak include lower hood length (LHL) and lower crest length (LCL) (Figure 1). Here, the lower crest length (LCL) was also used for lower squid beaks instead of the traditional measurement of Lower Rostral Length (LRL).

**FIGURE 2:**
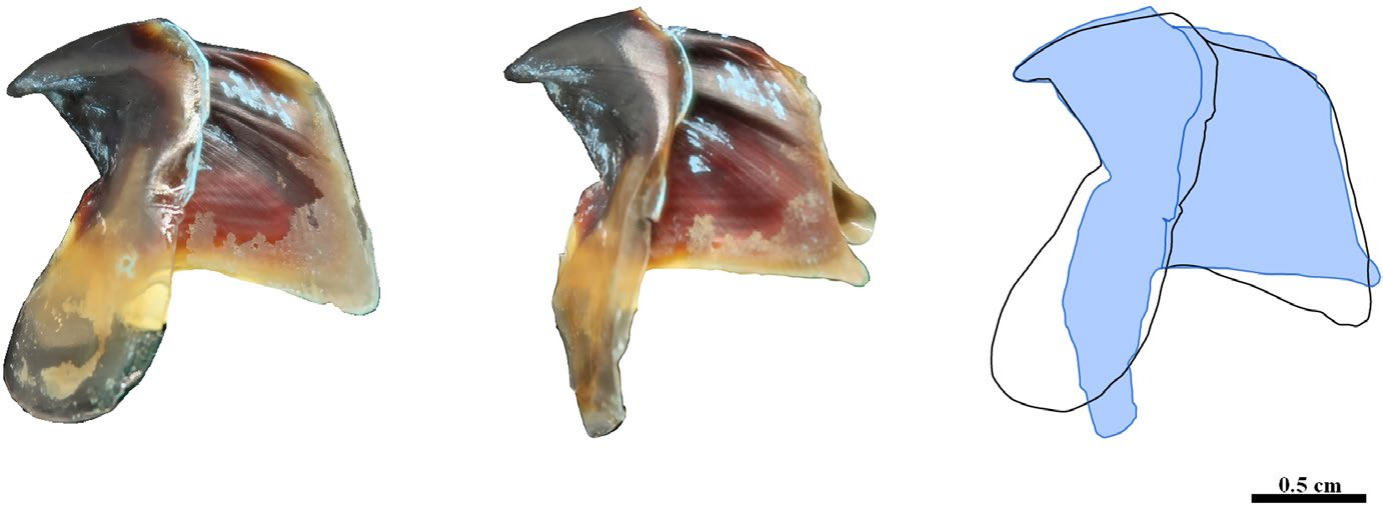
Deformation of a lower beak, here *Ommastrephes cylindraceus* as an example to illustrate the dramatic deformation due to dehydration

Cephalopod beaks change their shape when they become dehydrated (Figure 2) and cannot be imaged during the dehydration process or after. Consequently, using an underwater protocol is necessary to maintain the real configuration of these objects.

**FIGURE 3:**
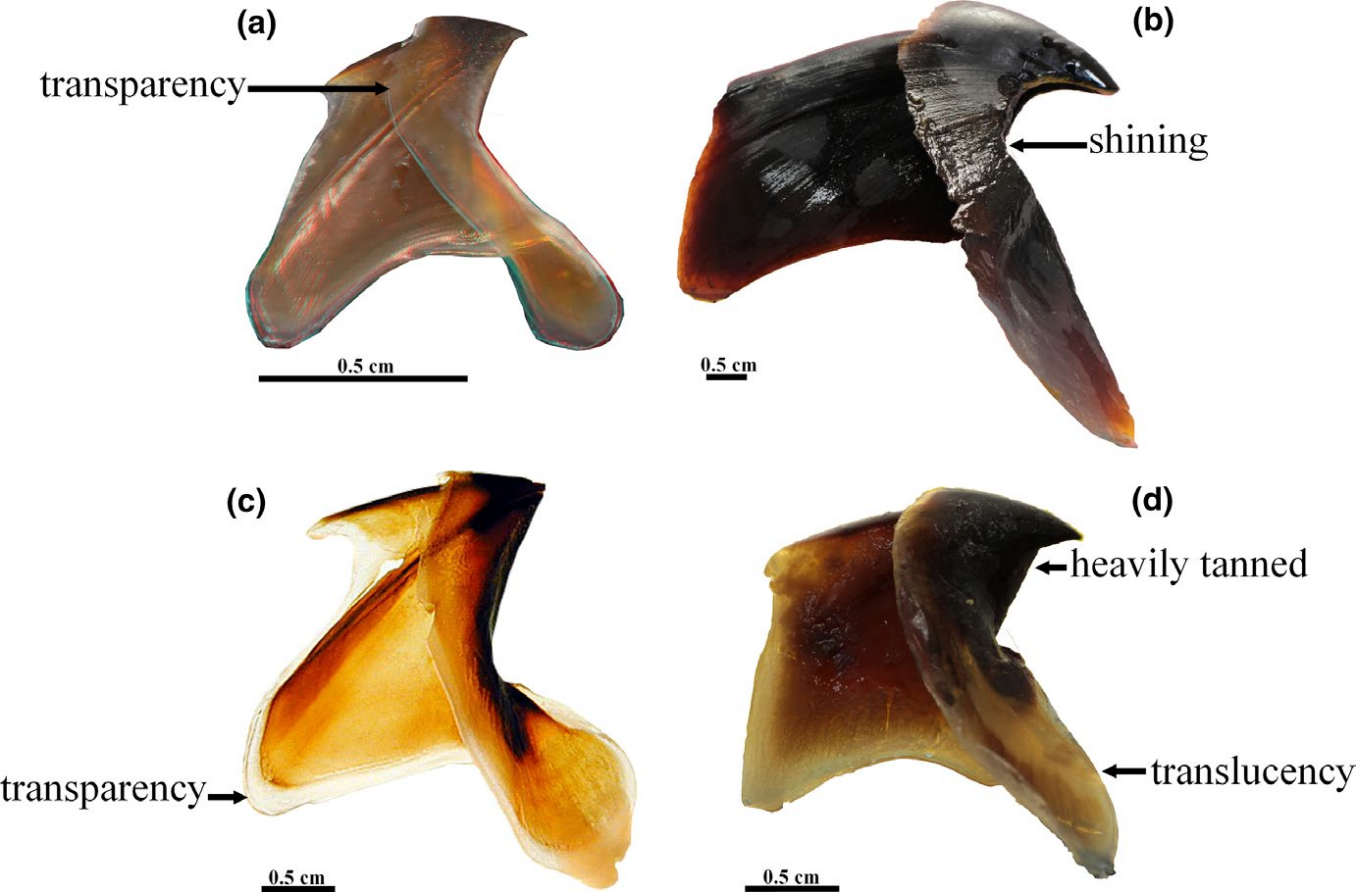
Examples of lower beaks of four species of cephalopods. A: *Mastigoteuthis psychrophila*, which is small and shows some transparency, B: *Sepia apama*, which has the largest and darkest beak, C: *Octopoteuthis* sp. with a mostly translucent and transparent beak with an extreme shape of the lateral walls, D: *Ommastrephes cylindraceus* presents a mixture of all possible difficulties related to photogrammetry including a considerable color gradient ranging from translucent to heavily tanned, is extremely thin and of intermediate size (around 17 mm)

In order to assess the accuracy of the underwater photogrammetry protocol described here, we selected four species of cephalopods that each present different levels of difficulty with regard to photogrammetry including their size, color, thickness, transparency, and surface reflection (Figure 3). *Mastigoteuthis psychrophila* Nesis, 1977 (Figure 3a) is the smallest beak (LCL = 5.37 mm) with some transparency that was successfully imaged in 3D using photogrammetry. *Sepia apama* Gray, 1849 is the largest (LCL = 50.77 mm) and the most tanned beak in our sample and it has an extremely dark and homogeneous texture (Figure 3b). *Octopoteuthis* sp. (Figure 3c) is mostly transparent and translucent and has extremely sharp and thin lateral walls which are particularly hard to capture on the pictures. *Ommastrephes cylindraceus* d'Orbigny, 1835 (Figure 3d) has a mixture of translucency, a heavily tanned rostrum, a global color gradation, and a smooth texture and is of intermediate size (LCL = 16.75 mm).

### 2.2 Photography material

**FIGURE 4:**
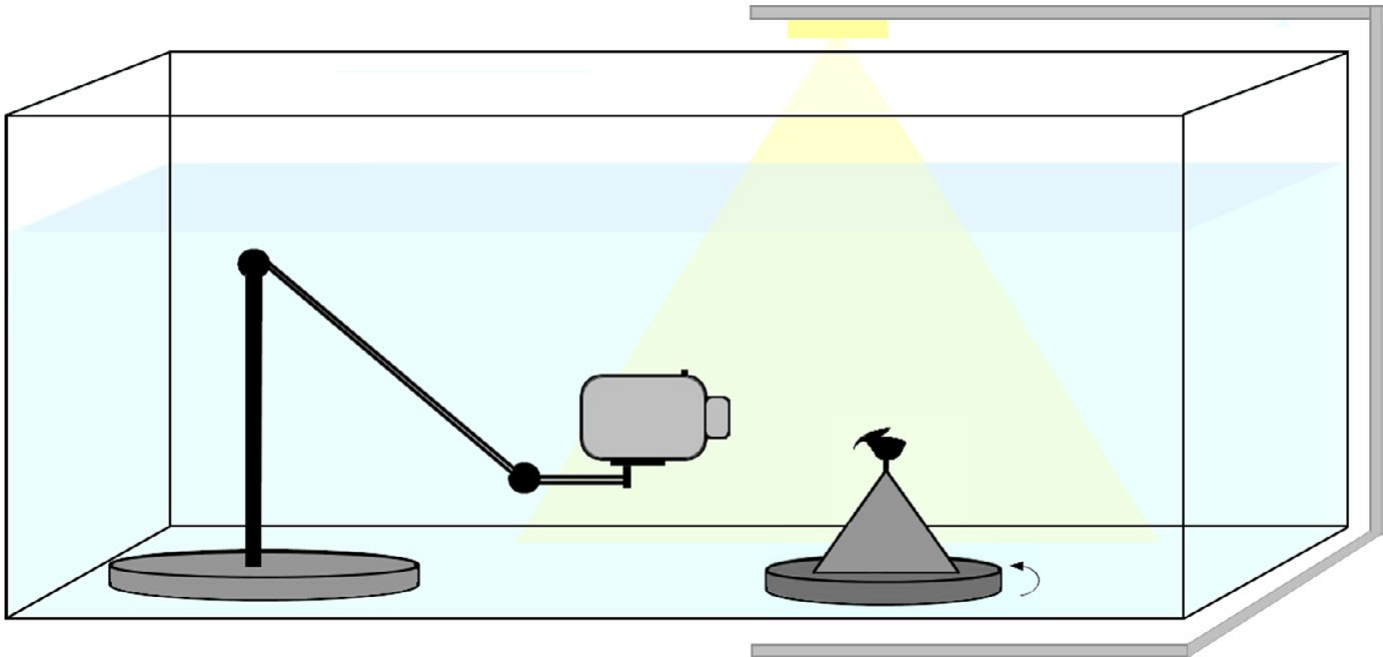
Photogrammetry setup with a tank full of water, led lights, a turning table, a pivoting arm, and a waterproof housing

The photogrammetry protocol was performed using a Canon Powershot G7X Mark II camera (focal length 8.8–36.8 mm) with a 15.2 mm focal length, focus F/3.5, and an Ikelite waterproof housing device with plate glass. The whole apparatus was fixed on a pivoting arm. This device is positioned in a tank illuminated with a set of white photographic studio led lights (Figure 4). The lighting setup is placed above the object, thus limiting shadow effects, and is diffused to homogenize the scene, avoiding reflections. The beak is fixed on a colored support on a turning table under water. Photogrammetry is performed under controlled conditions in the tank including homogeneous light and clear water. As the camera is equipped with a planar front window, which can be geometrically considered as an image‐invariant interface, no corrections for refraction were applied (Maas, 2015). Moreover, as we worked at 360 degrees around the object, the potential deformation is minimized and can be neglected.

### 2.3 Photo acquisition

As the beaks are small and thin, and have a uniform surface texture, it is difficult for the photogrammetry algorithm to identify points that can be tracked throughout the image sequence allowing image matching. Thus, we created reference points on the beak using physical marking (Luhmann et al., 2020).

For this purpose, each specimen is quickly extracted from the water to color parts with a water‐based removable paint pen. In this case, a blue POSCA was chosen to contrast with the beak color and the background. In addition, beaks are extremely fragile and the POSCA pen is easily removable with a toothbrush, avoiding damage to the beak. The most translucent parts are fully colored and the others are just covered with dots and stripes. A few seconds are needed to let the paint dry on the surface. Next, the beaks are fixed on a small support with removable decorative glue, here a hot‐glue that is easily detached from the beak when it dries, and inserted into a support fixed on an underwater turning table. Each picture is taken with underwater color parameters and in macro photography mode. These parameters enhance contrast and luminosity underwater and allow very close‐range pictures with a high depth of field to be taken. Two sets of pictures are taken for each beak in two different positions. This is done by attaching the beak by its dorsal surface, and then subsequently, by its ventral surface. This allows completing the model with the inside information and to avoid the loss of information because of the support. Photographs are taken each 5–10 degrees around the beak and from three different angles ranging from 0 to 45 degrees. Additional pictures are taken as close as possible to enhance details.

### 2.4 Image processing and reconstruction

**FIGURE 5:**
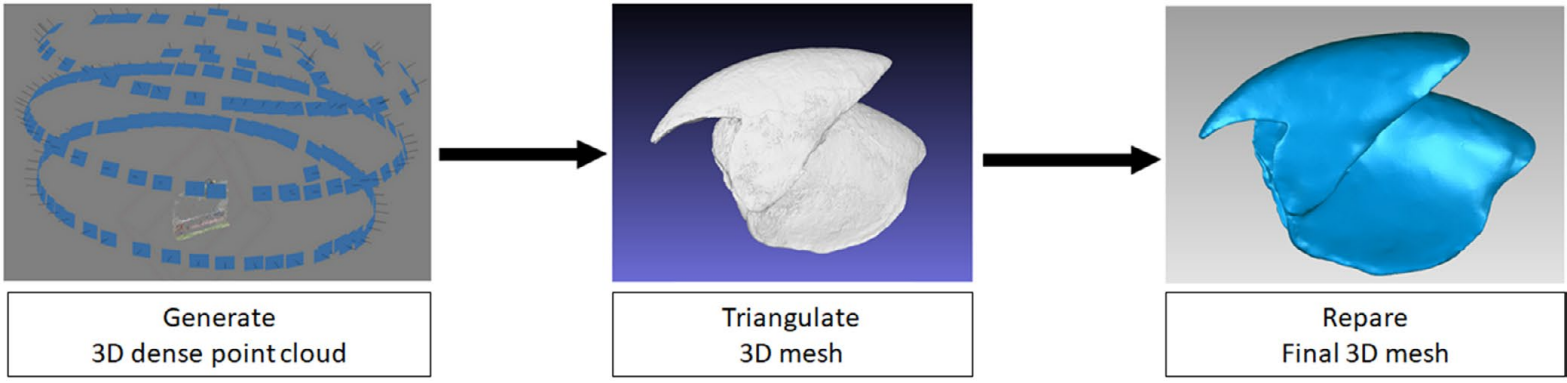
Summary of the steps followed using different types of software to obtain the final mesh. Each step can be achieved using either an open‐source software or not

To create dense point clouds that represent the structure of interest the imaging software Agisoft Metashape (Version 1.4.0) was used for the 3D reconstructions. Alternatively, open‐source software packages such as Meshroom can be used to generate these dense point clouds. Two different dense point clouds of each beak are obtained corresponding to the two acquisitions, one for each side of the beak. Both dense clouds were then cleaned and markers were selected. Next, using the markers as reference, clouds are aligned and matched to reconstruct the whole object. However, it was not possible to triangulate meshes with the software algorithm because of the narrow walls of the beak. Indeed, smoothing iteration and Delaunay spherical diameter reconstructions did not allow capturing the thickness of the beak without any loss of information. The thickness of the beak is so reduced that it only represents a surface and not a volume at its thinnest parts. To avoid the loss of points and the creation of holes during mesh processing, the open‐source software MeshLab (version 2016.12) was used to triangulate the dense point cloud (Cignoni et al., 2008) produced by Agisoft Metashape. The Screened Poisson Surface reconstruction algorithm from Kazhdan and Hoppe (2013) was used to triangulate models and does not contain smoothing steps. However, the obtained mesh is quite noisy resulting in the need to remove some artifacts and the filling of remaining holes. Smoothing and closing of holes were performed with Geomagic Studio (Version 2013.0.1.1206). As Geomagic Studio is not an open‐source software, it is possible to use other alternatives such as CloudCompare, Autodesk MeshMixer, or 3D Slicer to repair small imperfections of the mesh. We scaled each dense cloud in Metashape using a scale bar positioned under the specimen and by applying scaling to the reconstruction. We measured our obtained 3D model in MeshLab to compare it with the dimensions of the real object (Figure 5).

### 2.5 Accuracy of the photogrammetry protocol

In order to compare the models obtained by photogrammetry to an intact undeformed model, an upper and a lower beak of the squid *Ommastrephes cylindraceus* were scanned at the micro‐CT scan facilities at the National Museum of Natural History in Paris (AST‐RX) using GE phoenix v|tome|x L 240‐180 micro‐CT. Beaks were scanned with the microfocus high‐resolution X‐ray tube at 60 kV and 360 mA, without filter and at a voxel size of 19 mμ. Beaks were immobilized in a 1% agarose gel to avoid deformation or movement during scanning. The model was reconstructed using Materialise Mimics (v.21.0). After the segmentation of the micro‐CT scan data, some holes may remain in the 3D model. To fully represent the surface, the holes were filled in Geomagic Studio as done for the photogrammetric model. Then, both 3D models were compared.

To quantify the methodological error created using photogrammetry and tomography protocols, respectively, we performed a Student *t* test on the mean distances between a sample of thirty‐seven upper beaks and thirty‐six lower beaks, half of them acquired using photogrammetry and half of them acquired through micro‐CT scanning at the National Museum of Natural History in Paris (AST‐RX) using a GE phoenix v|tome|x L 240‐180 micro‐CT and at the National History Museum in London using Zeiss Xradia Versa 520 micro‐CT (Table S1). First, we used MeshLab to align each beak on a principal axis, in order to fit it into a 3D virtual box. Next, the meshes were imported in R (version 4.0.2) (R Core Team, 2020). Each pair of beaks was then scaled using the box size and realigned on the principal axis. Meshes were then aligned and the distance between both meshes was computed (see meshDist function in Schlager (2017)). Mean distances were used to test whether these distances are significantly greater than the mean distance between the two methods, calculated for the *Ommastrephes cylindraceus* beaks. This test was performed in order to ensure the absence of a bias due to the acquisition method (CT versus photogrammetry) when including closely related species (Table S1).

To represent the differences between both methods, a best‐fit alignment was performed in Geomagic. The best‐fit algorithm performs an alignment of a sample of random points of the test mesh onto the reference until the distance is beyond the tolerance. Then, the entire mesh is aligned with the reference mesh (Geomagic User guide). When the distance between both models is the smallest, the 3D compare function of Geomagic calculates poi

nt distances and generates a deviation map.

## 3 RESULTS

**FIGURE 6:**
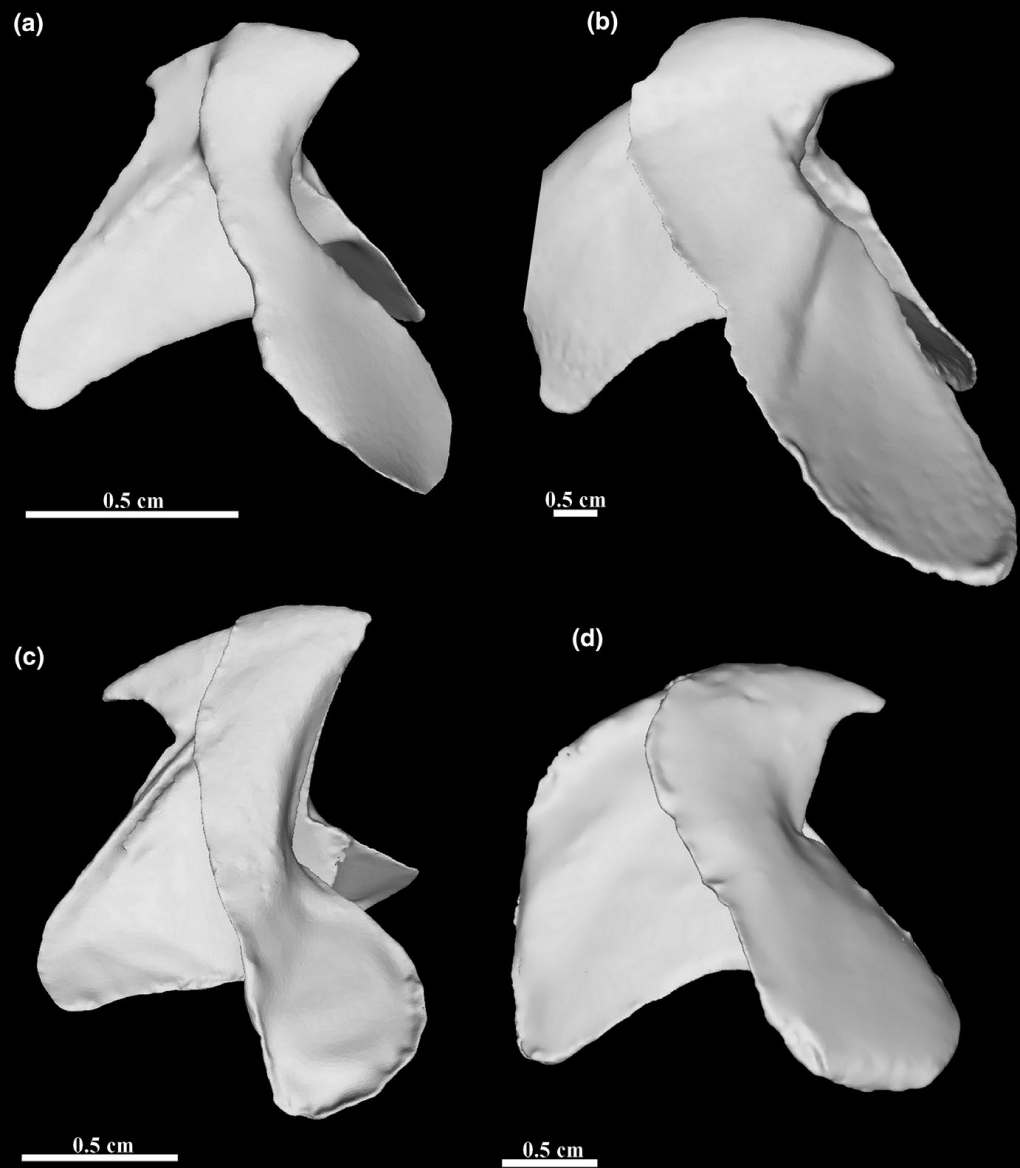
Lateral view of 3D photogrammetric models of cephalopod beaks. Illustrated are lower beaks oriented with the anterior to the right and the ventral side up. A: *Mastigoteuthis psychrophila*, B: *Sepia apama*, C: *Octopoteuthis* sp., D: *Ommastrephes cylindraceus*

All the beaks captured with the photogrammetry protocol were fully reconstructed and their measurements and proportions are similar to those of the real objects (Table 1). The largest beak we reconstructed is the heavily tanned lower beak of *Sepia apama* (Figure 6b). It is thicker than all the others beaks but its homogeneous texture prevented a straightforward reconstruction. With the POSCA marker added, especially at the edges, the beak became fully textured and easy to reconstruct. The lower beak of *Octopoteuthis* sp. was reconstructed correctly using our protocol despite the unusual structure of the lateral walls (Figure 6c). The walls of the beak in this species are extremely short and thin at the crest and they create a large opening. Most of the extremities of the walls are translucent. Moreover, they show folding, a used morphological features for lower beak identification. Using the marker, the macro photography mode, and detailed pictures, we were able to fully capture its shape. The *Ommastrephes cylindraceus* (Figure 6d) lower beak represented multiple difficulties. It has a translucency gradient from translucent at the edges of the beak to heavily tanned on the rostrum. It is of intermediate size (LCL around 17 mm) but it is thin and not easy to capture. Finally, the smallest beak that was reconstructed was the lower beak of *Mastigoteuthis psychrophila* with a lower crest length (LCL) of around 5 mm (Figure 6a). It is also the thinnest and the most difficult object reconstructed. Whereas typically 300 pictures were used for a reconstruction, this beak required 350 pictures and more close‐up pictures to be able capture the tiny crest and hood.

**TABLE 1:** Comparison of measurements between lower beaks and their 3D models

Species	LHL (mm)	LHL model (mm)	LCL (mm)	LCL model (mm)	Nb photos
*Mastigoteuthis psychrophila*	2.81	2.83	5.37	5.01	360
*Sepia apama*	23.28	23.71	50.77	51.30	325
*Octopoteuthis* sp.	6.98	7.09	15.34	15.76	302
*Ommastrephes cylindraceus*	9.00	8.91	16.75	16.02	299

Abbreviations: LCL, Lower Crest Length; LHL, Lower Hood Length.

Measurements on real jaws were performed with calipers, and the rule tool was used in MeshLab for 3D models. Measurements of cephalopod beaks taken from the 3D models (LCL and LHL) are similar to those of real jaws (Table 1) but models appear slightly larger as observed for *Sepia apama* and *Octopoteuthis* sp. or slightly smaller as for *Mastigoteuthis psychrophila* and *Ommastrephes cylindraceus*. Differences can be due to a global increase in the thickness of the beak models or an underestimation of the length at the end of the crest. Yet, for the four specimens, the difference in lower hood length (LHL) is less than 2% of the size measured on the specimens. The crest length shows more difference, up to 6.7% in *Mastigoteuthis psychrophila* but as it is very small, the caliper measures may be more erroneous than the measures on the 3D model. The extreme parts of the crest were sometimes hard to capture because of its transparency and may as such result in a slight underestimation of the crest length, as for *Ommastrephes cylindraceus*.

**FIGURE 7:**
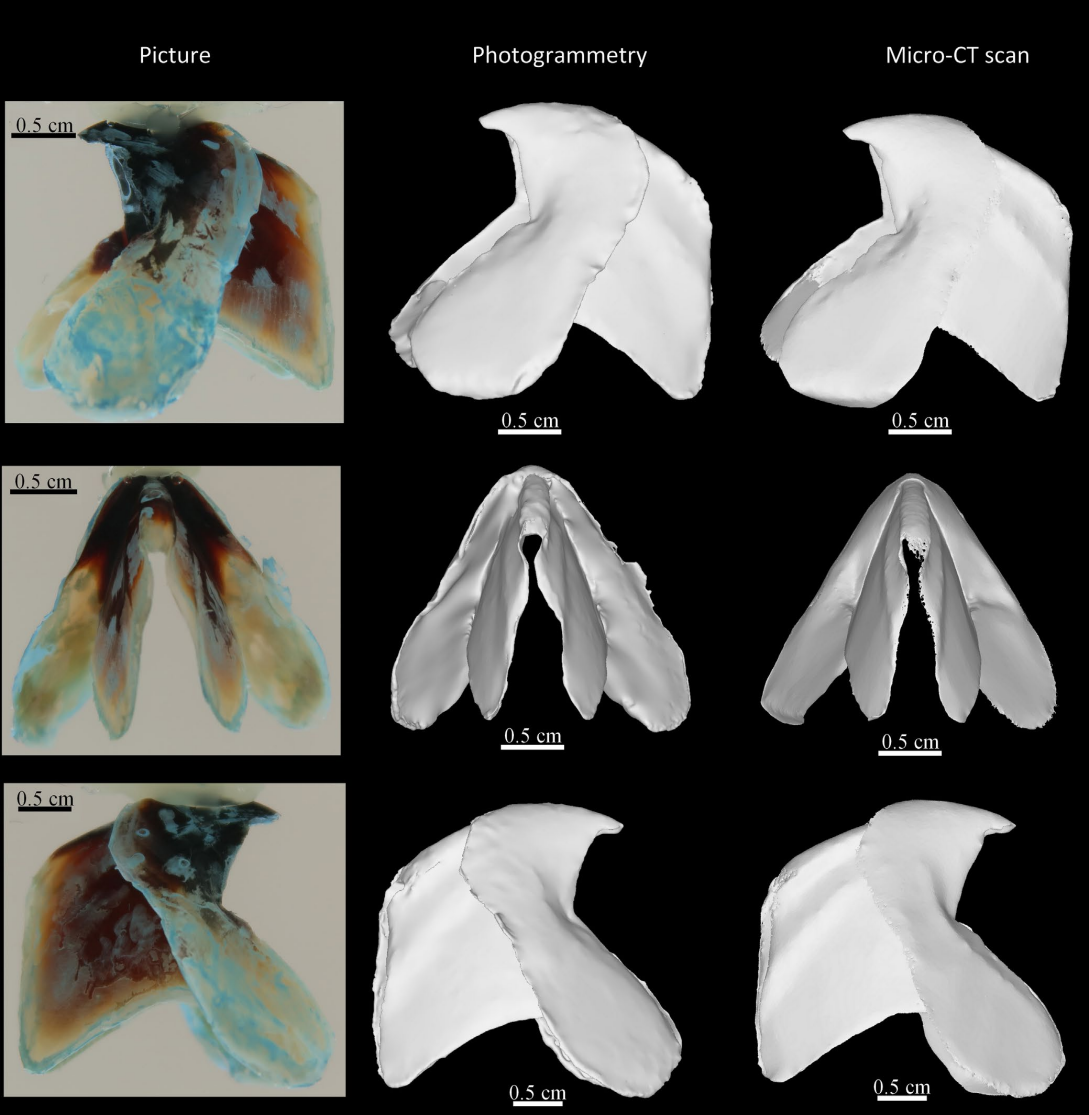
Comparison between photogrammetric model of a lower beak of *Ommastrephes cylindraceus* and the same reconstruction using micro‐CT scanning. top: lateral right view; middle: posterior view; bottom: lateral left view. Pictures conserve the conventional orientation of beaks

**FIGURE 8:**
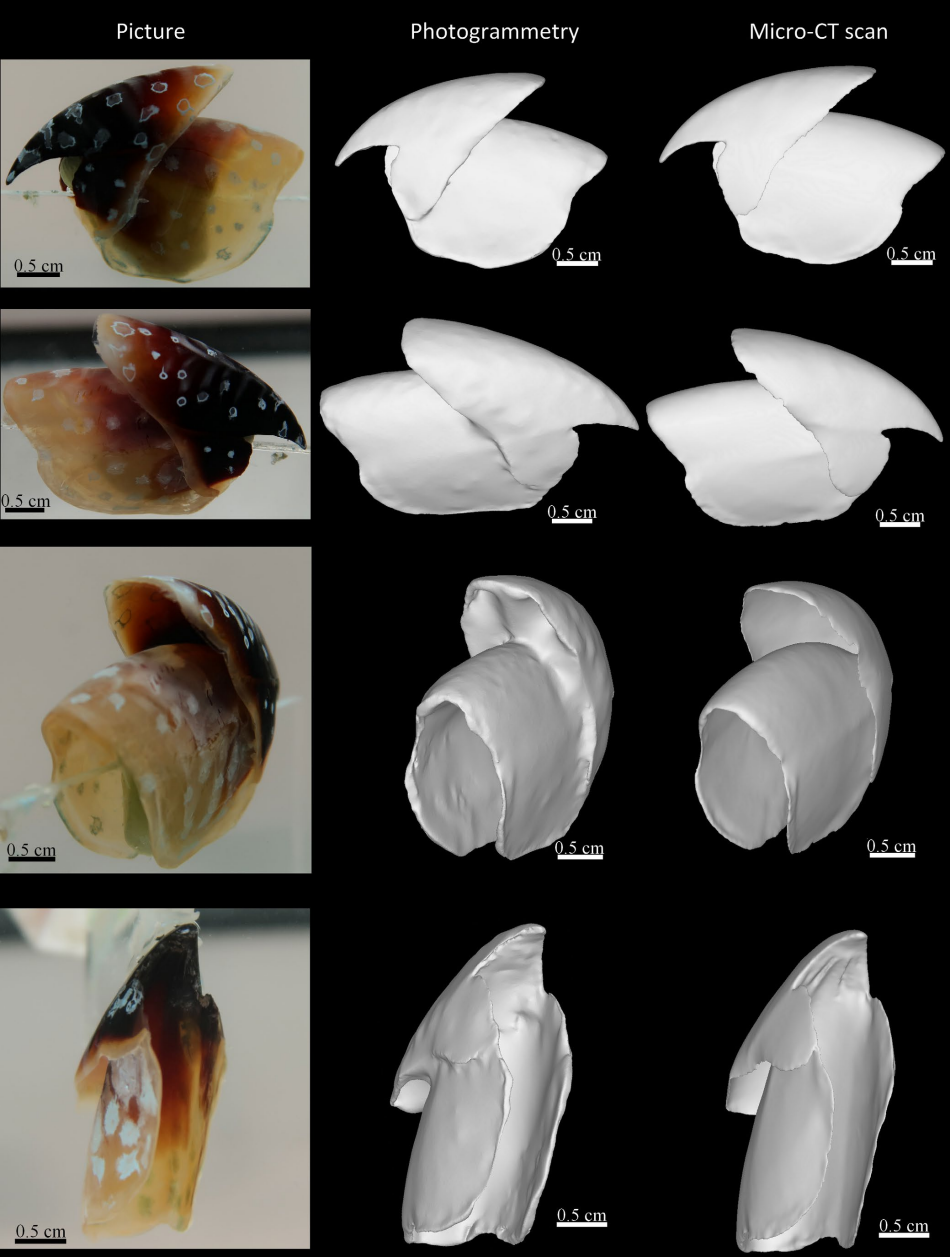
Comparison between a photogrammetric model of an upper beak of *Ommastrephes cylindraceus* and the same reconstruction using micro‐CT scanning. top: lateral left view; middle: lateral right and posterior views; bottom: ventral view. Bottom picture is upside‐down to conserve the conventional orientation of beaks

To examine the accuracy of the photogrammetric model, we compared the reconstruction of a lower beak (Figure 7) and an upper beak (Figure 8) of *Ommastrephes cylindraceus* using the described photogrammetry protocol with a 3D model obtained through micro‐CT. Although both protocols represent the global shape of the beaks well, photogrammetry is somewhat noisier. On both beaks, the lateral walls and wing surfaces are less smooth for the models obtained with photogrammetry than after micro‐CT scanning. However, some irregularities are better captured with photogrammetry, particularly on the thin tips of the wings and lateral walls. These extremities can be difficult to reconstruct in detail when segmenting the CT images and when smoothing is applied as the fully transparent edges of the beak may be extremely difficult to identify in the scans. The inner part of the hood is not accessible with photogrammetry because of its structure and concavity. Indeed, a portion of the hood fully covers the inner part of the walls and is difficult to capture in photographs. For these reasons, this part of the beak morphology is much easier to reconstruct using micro‐CT scans. The general shape of both models is similar and models have similar measures and proportions compared with real beaks. The extremely thin part of the posterior crest of the lower beak is not correctly reconstructed in both cases. This may be due to the fact that this part is transparent and thus difficult to identify both on pictures and scans.

**FIGURE 9:**
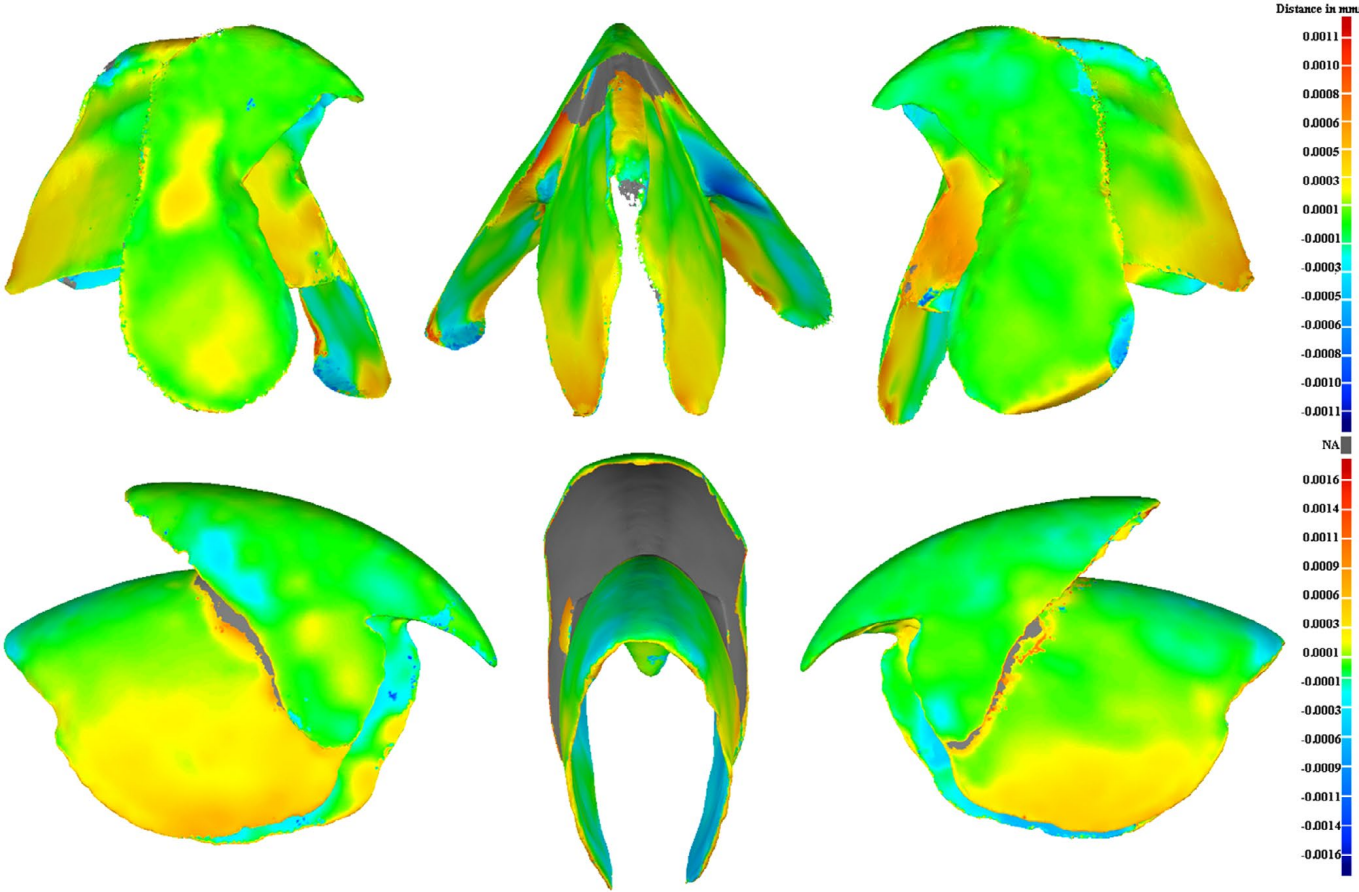
Distance map computed with Geomagic between micro‐CT models and the 3D models *Ommastrephes cylindraceus* reconstructed by photogrammetry. Top: Lower beak, Bottom: Upper beak. The grey parts are not assessed because they could not be imaged using the photogrammetry protocol

The differences between both models (CT vs. photogrammetry) estimated using the best‐fit alignment in Geomagic are small. Most of the parts are identical and the maximum deviation is about 0.001 mm, on some of the inner parts or the extremities (red and deep blue parts; Figure 9). Models reconstructed with photogrammetry are somewhat thicker and less regular than those obtained by micro‐CT scanning, but differences are small and should not impact the use of the models for species identification or even geometric morphometric analyses (Zelditch et al., 2012).

**FIGURE 10:**
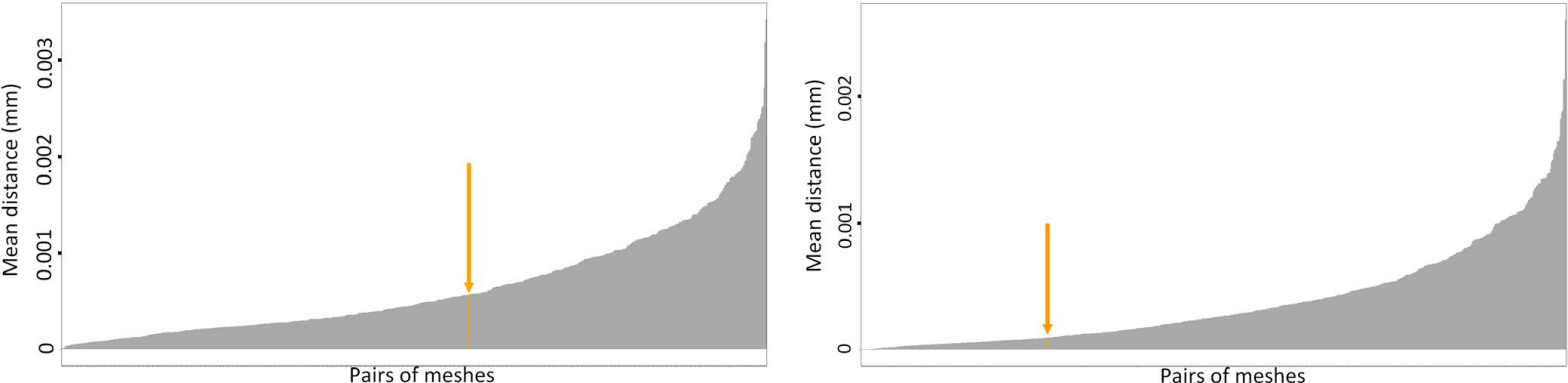
Mean distances between pairs of meshes for upper beaks (top) and lower beaks (bottom). Orange value is the mean distance between the same model using both methods, and mean distance is 9.47·10^−5^ mm for upper beaks and 5.66·10^−4^ mm for lower beaks

To quantify the differences between both methods with the shape variation between two specimens of the same species and closely related species, we computed the mean distances between pairs of thirty‐seven lower and thirty‐six upper beaks. Figure 10 shows the mean distances between these shapes for both upper and lower beaks. The orange bar represents the mean distance between both methods computed with *Ommastrephes cylindraceus*. It shows that the distance between the two methods is similar to the smallest values of the different model comparisons. A unilateral Student's *t* test confirmed that mean distances between pairs of beaks are significantly greater (*t* = 32.202, *p* < 2.2e−16 for upper beaks, *t* = 56.958, *p* < 2.2e−16 for lower beaks) than the distance between both methods.

## 4 DISCUSSION

Many natural objects that are deformable, dry‐sensitive, thin, smooth, or with a significant transparency gradient (i.e., chitinous skeletons of arthropods, cartilage, some plant tissues) cannot be included in studies requiring large samples of 3D morphological data because the acquisition of 3D models remains technically challenging or expensive. In these cases, the use of underwater photogrammetry could meet these needs. However, there are still limitations and difficulties associated with the underwater imaging of specimens with uniformly colored, smooth, transparent, and thin features. The aim of this study was to present an adapted underwater microphotogrammetry protocol to reconstruct accurate 3D models of objects combining multiple challenges including size, transparency, and dry sensitivity. Cephalopod beaks are used here as an ideal example as they present numerous difficulties (size, shape, a chitinous texture, transparency gradient) for photogrammetry and micro‐CT scanning. For these reasons, no database of 3D models for species identification or to study morphological variation or function has been published to date.

Accurate 3D modeling of beaks exhibiting variation in size, transparency, and thickness was possible by marking the surface of the beak, together with the use of a camera with a macro photography mode allowing a considerable depth of field and a bright homogeneous light. Depending on the combination of these different issues for each species of cephalopods, more or fewer pictures, especially more detailed pictures, were needed. The density of the point clouds may be variable depending on the number and quality of the pictures but the triangulated mesh was almost always wrong when using the commercial Agisoft Metashape software. Edges disappeared and holes were created everywhere in the model, such that the overall shape was not conserved. The use of MeshLab and then Geomagic to fully triangulate and remove artifacts of beaks was necessary, yet more time‐consuming. After the reconstruction of the dense point cloud (the duration of which depending on the computer used), we estimate that we needed a mean of 2 hr to complete the model and to be able to use it for further analyses. In contrast, the sample preparation in agar, scanning time, segmentation and fully completing a model derived from micro‐CT scanning represents roughly 8 hr of work. Unfortunately, automatic thresholding of CT scans cannot be used to capture the shape of the beak because it provides little contrast and the less tanned, or fully untanned parts need to be reconstructed manually. This probably explains the smoothness of the edges of the *Ommastrephes cylindraceus* lower beak compared with the reality of the noisy edges, as illustrated with photogrammetry.

Despite some small differences, both models captured the overall measurements of the beaks well. We noticed that the smallest beaks had the largest crest length discrepancies. This is probably due to a combination of a less accurate caliper measure as the beak is extremely fragile, together with the difficulty of reconstructing the 3D model. In contrast, the error for *Ommastrephes cylindraceus* beaks was likely due to the less accurate representation of the extreme part of the crest which is too translucent. However, this does not impact specimen identification based on the 3D models only. In summary, 3D models produced by photogrammetry correctly described the beak shape except for the inner part of the hood, which could not be reconstructed in 3D. As this part is not used for identification, this photogrammetry protocol can at least be used for taxonomic studies.

Three dimension models, whether derived from photogrammetry or tomography, are always an interpretation of real objects. A lot of methods have been compared with photogrammetry, such as CT scanning, (Giacomini et al., 2019; Hussien et al., 2019), laser scanning (Baltsavias, 1999; Gibelli et al., 2018), surface scanning (Fau et al., 2016) and its accuracy and precision has been demonstrated (Bythell et al., 2001; De Menezes et al., 2010; Figueira et al., 2015; Varón‐González et al., 2020). Here, we show that differences between models produced by photogrammetric means and micro‐CT are small and should not impact species identification or morphometric analyses. Moreover, some minor features of the lateral walls (i.e., ridges or folds, which are thickened, or crests on lateral walls as seen on *Mastigoteuthis psychrophila* and *Octopoteuthis* sp.) are smoother with photogrammetry than after micro‐CT scanning, but they are still present. Furthermore, photogrammetry allowed us to capture small irregularities on the finest parts of the beaks even if they are transparent, whereas these can be extremely hard to see on the CT scans. Although some parts are more difficult to capture with our compact camera (e.g., the inner part of the hood), professional photographic equipment, such as a digital SLR camera, a macro lens, and better lighting, would allow to get a higher resolution and capture these parts with a greater accuracy. However, the thickness, transparency, and fragility of the beaks appear to be true limitations of this protocol.

Despite some flaws in our photogrammetry models such as the areas not captured, the 3D models obtained can be used to analyze the shape of the beak with an accuracy that 2D pictures do not provide. Indeed, beaks have a complex shape with a different width on the anterior and on the posterior parts; they may have ridges or folds on their lateral walls and their global shape provides information on muscle attachment. All these characteristics cannot be captured with two‐dimensional images and analysis, yet are critical if the aim is to identify species or to study beak function. Indeed, 3D models are essential to study the unusual articulation and function of the beaks (Uyeno & Kier, 2005) and their ontogeny (Franco‐Santos & Alves Gonzalez Vidal, 2020; Uchikawa et al., 2009) or to extract information on trophic level (Golikov et al., 2019; Staudinger et al., 2019). As texture maps can be overlaid onto photogrammetrically derived 3D models, it will also be possible to create a 3D catalogue of beaks for identification. (Xavier & Cherel, 2009; Young et al., 2019). Finally, these models can help to reference some rare and poorly known beaks that are represented by only few specimens in collections without damaging them.

Whereas high‐resolution micro‐CT scanning is more time‐consuming and expensive, photogrammetry allowed us to reconstruct a representative model of different cephalopod beaks with portable material and an easy‐to‐use and fast protocol. Other deformable and easily dehydrated and sensitive objects such as shells, cartilage, or chitinous structures (i.e., thin and translucent exoskeletons) of insects, amphipods, and copepods might also be accurately reconstructed with this method. We further show that thin structures can also be imaged, offering opportunities for broad comparative 3D studies for bivalve shells for example (Scalici et al., 2016), suggesting that our protocol has a broad application in reconstructing 3D objects for studies in ecology and evolution.

## CONFLICT OF INTEREST

None declared.

## AUTHOR CONTRIBUTIONS

**Marjorie Roscian:** Conceptualization (lead); formal analysis (lead); methodology (lead); visualization (lead); writing–original draft (lead). **Anthony Herrel:** Project administration (equal); supervision (equal); validation (equal); writing–review and editing (equal). **Raphaël Cornette:** Validation (equal); writing–review and editing (equal). **Arnaud Delapré:** Validation (equal); writing–review and editing (equal). **Yves Cherel:** Resources (equal); writing–review and editing (equal). **Isabelle Rouget:** Project administration (equal); supervision (equal); validation (equal); writing–review and editing (equal).

## Supporting information

Table S1

## Data Availability

Scans from computed tomography are available in the archive of the AST‐RX platform in Paris and in National History Museum imaging archive in London, UK. Table S1: List of specimens used for the computation of mean distances can be found in DRYAD : https://doi.org/10.5061/dryad.4mw6m9095.
